# Adjuvant-free, self−assembling ferritin nanoparticle vaccine coupled with influenza virus hemagglutinin protein carrying M1 and PADRE epitopes elicits cross-protective immune responses

**DOI:** 10.3389/fimmu.2025.1519866

**Published:** 2025-01-31

**Authors:** Yongqiang Zhao, Shuangshuang Guo, Jia Liu, Yating Wang, Bo Wang, Chun Peng, Enqi Du

**Affiliations:** ^1^ College of Veterinary Medicine, Northwest Agriculture and Forestry (A&F) University, Yangling, Shaanxi, China; ^2^ Department of Research and Development, Yangling Carey Biotechnology Co., Ltd., Yangling, Shaanxi, China; ^3^ Department of Research and Development, Chengdu NanoVAX Biotechnology Co., Ltd., Chengdu, Sichuan, China

**Keywords:** influenza virus, nanoparticles, adjuvant-free, cross-protection, vaccines

## Abstract

**Introduction:**

Influenza viruses pose a significant threat to global public health. Several influenza pandemic outbreaks have had serious economic and public health implications. Current influenza virus vaccines generally provide strain-specific protection and must be rapidly produced annually to match the circulating viruses. Developing influenza vaccines that confer protection against a broad range of viruses will have a positive impact on public health. In this study, we aimed to develop a ferritin-based influenza nanoparticle vaccine with a broad protective spectrum to enhance the immune response against diverse influenza viruses.

**Results:**

We generated an adjuvant-free, self-assembling nanoparticle vaccine against diverse influenza A viruses. This nanoparticle vaccine displayed multi-antigen targets on the surface of *Helicobacter pylori* ferritin, which consists of the ectodomain of hemagglutinin of the H3N2 virus and three tandem highly conserved influenza M1 epitopes fused with the universal helper T-cell epitope PADRE, named HMP-NP. HMP-NPs were expressed in a soluble form in the baculovirus-insect cell system and self-assembled into homogeneous nanoparticles. Animal immunization studies showed that the HMP-NP nanovaccine elicited 4-fold higher haemagglutination inhibition (HAI) titers than inactivated influenza vaccine. And neutralization titers induced by HMP-NPs against the H3N2 virus and heterologous strains of the H1N1 and H9N2 viruses were ~8, 12.4 and 16 times higher than inactivated influenza vaccine, respectively. Meanwhile, we also observed that the number of IFN-γ- and IL-4-secreting cells induced by HMP-NPs were ~2.5 times higher than inactivated influenza vaccine. Importantly, intranasal immunization with HMP-NPs, without any adjuvant, induced efficient mucosal IgA responses and conferred complete protection against the H3N2 virus, as well as partial protection against the H1N1 and H9N2 viruses and significantly reduced lung viral loads.

**Discussion:**

Overall, our results indicated that the self-assembled nanovaccines increased the potency and breadth of the immune response against various influenza viruses and are a promising delivery platform for developing vaccines with broader protection against emerging influenza viruses and other pathogens.

## 1 Introduction

Influenza is a highly contagious respiratory infection and continues to pose health and economic burdens (1, 2). Seasonal influenza causes over 300,000 deaths annually (3). Current seasonal influenza vaccines contain three or four circulating influenza virus strains. The trivalent vaccine covers circulating strains of influenza A virus subtypes H1N1 and H3N2 and the Victoria lineage of influenza B virus. In addition to these strains, the quadrivalent vaccine also protects against the Yamagata lineage of influenza B virus. These vaccines are effective when they match well with the circulating strains. However, the composition of vaccine strains relies on annual predictions by the World Health Organization (WHO), and mismatches occur relatively frequently (4–6). Importantly, seasonal influenza vaccines are ineffective against emerging strains in the case of pandemic outbreaks. Therefore, the development of a universal influenza vaccine that elicits cross-protective immune responses against divergent influenza virus strains is essential for future influenza prevention and control strategies.

Influenza virus hemagglutinin (HA) is the major surface antigenic glycoprotein responsible for binding viruses to infected cells. Most current influenza virus vaccines target the immunodominant head domain of HA, which makes them strain-specific (7). HA-induced antibodies often have neutralization and hemagglutination inhibition (HI) activities and can bind to the virus early in the course of infection and inhibit viral invasion into cells (8). Unfortunately, the head domain of HA is highly susceptible to mutations (9), and the design of a more optimized vaccine is critical to avoid this problem.

Many efforts have focused on developing universal vaccines that confer broader protection against diverse influenza viruses, including by targeting conserved viral proteins (5, 10). These include the headless hemagglutinin (HA) stalk and matrix protein 2 ectodomain (M2e) (11–14). Matrix protein 1 (M1) is often highly conserved in the influenza virus (15). Since the M1 protein is generally present within the virus rather than on its surface, rendering it not easily accessible to immune surveillance. However, M1 proteins are produced in high quantities within virus-infected host cells, where they are processed and presented on major histocompatibility complex molecules to T cells (16). Targeting M1 proteins may therefore serve as an effective strategy for activating cytotoxic T lymphocytes (CTLs) to combat the infection. For this reason, another widely used target for universal influenza vaccines is the internal influenza virus M1 protein, which is mainly used as a target for a universal T cell–based vaccine that can induce cross-protection against heterologous influenza viruses (17–19).

However, the low immunogenicity of peptides hinders the development of recombinant protein- and peptide-based vaccines. One strategy for increasing the efficacy of these vaccines is to enhance the CD4^+^ T helper cell immune response. Consequently, to achieve effective immunization, the development of an immunization regimen that reliably induces the production of antigen-specific CD4^+^ T cells is crucial. Pathogen-derived peptides are recognized by T cell receptors (TCR) that drive antiviral CD4^+^ T cell-mediated immunity. In 2012, Wilkinson et al. demonstrated that CD4^+^ T-cell responses targeting conserved proteins of the influenza virus were associated with heterosubtypic protection against pandemic influenza A virus (IAV) (20). Greenshields-Watson et al. identified several influenza epitopes from internal proteins recognized by CD4^+^ T cells. Among these epitopes, the most immunogenic epitope, M1_129-142_, with the amino acid sequence GLIYNRMGAVTTEV, was able to stimulate an increased production of cognate CD4^+^ T cells in culture and showed stronger avidity for human leukocyte antigen (HLA) multimers across all donors compared to the well-studied HLA class II influenza epitope HA_306-318_-PKY (21). Additionally, a universal T-helper Pan HLA-DR epitope (PADRE) peptide, with the amino acid sequence AKFVAAWTLKAAA, binds to the majority of MHC class II molecules with high affinity (22). PADRE peptide-conjugated antigens have been used to improve the immunogenicity of vaccines in preclinical models (22–24).

Another strategy to enhance the immunogenicity of recombinant protein/peptide-based vaccines is to utilize a highly efficient antigen delivery system. Advances in nanobiotechnology have led to the development of platforms for vaccine delivery (25–29). Many naturally occurring proteins self-assemble into nanoparticles, such as ferritin, an intracellular iron storage protein that self-assembles into nearly spherical nanoparticles (30, 31). Self-assembled ferritin is well-suited to display antigens of interest at a high density on its surface by gene fusion, thereby enhancing antigen immunogenicity. Ferritin nanoparticle platforms have been extensively used to display antigens from various viruses, including influenza viruses, respiratory syncytial virus, HIV, and SARS-CoV-2, and they elicit more potent and effective immune responses than soluble antigens (32–37). In recent years, many ferritin-based influenza nanoparticle vaccines that induce cross-protection against divergent influenza viruses have been developed (26, 34, 38, 39). Therefore, self-assembled ferritin could serve as a potential protein nanoplatform for antigen delivery, presentation, and immunostimulation, contributing to the induction of efficient immune responses and enhancing vaccine efficacy.

Given that influenza viruses invade the body through mucosal surfaces, which serve as the first line of defense against pathogen invasion, it is important to consider the role of the mucosal immune system. This system is not only a critical component of the overall immune network but also functions as an independent immune system with distinct structures and functions. Nasal vaccines have the capability to induce both mucosal and systemic immune responses, thereby providing immunoprotective benefits during the early stages of pathogen invasion and effectively addressing the limitations of existing vaccines. Intranasal immunization is recognized as a safe and effective method for inducing mucosal immunity and combating influenza virus infection. Pervious studies have indicated that nasal immunization provided cross-protection against diverse viral infections by eliciting highly effective local mucosal immune responses in the respiratory tract, along with robust humoral and cellular immune responses (25, 26). In our research, we established two administration routes for immunization: intranasal and intramuscular. We compared the immunization effects of these two routes.

In this study, we developed a ferritin-based influenza nanoparticle vaccine with a broad protective spectrum to enhance the immune response against diverse influenza viruses. The ectodomain of A/Darwin/6/2021 (H3N2) HA, three repeated M1 epitopes, and the T-Helper Pan HLA-DR epitope PADRE were tandemly fused to the N-terminus of ferritin. The fusion protein was produced using a baculovirus-insect cell system and denoted as HMP-NP. Without an additional adjuvant, immunization with influenza HMP-NPs induced highly potent, broadly protective immunity and conferred complete protection against lethal challenges by the H3N2 virus and partial protection against H1N1 and H9N2 viruses. These findings provide proof-of-concept for HMP-NPs as next-generation influenza vaccine candidates.

## 2 Materials and methods

### 2.1 Mice, cells, and viruses

Female specific pathogen-free (SPF) BALB/c mice aged 6–8 weeks were purchased from Xi’an Yifengda Biotechnology Co., Ltd. (Xi’ an, China) and maintained in individually ventilated cage (IVC) systems (Fengshi Group, Suzhou, China).

Madin-Darby Canine Kidney (MDCK) cells were maintained in Dulbecco’s modified Eagle’s medium (DMEM, Cytiva, USA) containing 10% fetal bovine serum (FBS; Gibco, USA), 100 U/mL penicillin, and 100 μg/mL streptomycin at 37°C with 5% CO_2_. Sf9 and Hi5 cells were cultured in IB905 serum-free medium (Yishengke, China).

A/Puerto Rico/8/34 (PR8, H1N1), H3N2 (CVCC AV1520), and A/Chicken/Jiangsu/7/2002 (H9N2) were cultured in SPF 10-day-old embryonic chicken eggs (Boehringer Ingelheim Witte Biotechnology Co., Ltd., Beijing, China), and the viruses were obtained from the allantoic fluid of the embryonic chicken eggs after 48 h. The 50% lethal dose (LD50) of the viruses was calculated using the Reed–Muench method. The swine virus strain H3N2 (CVCC AV1520) was obtained from the Center for Veterinary Culture Collection of China (China Institute of Veterinary Drug Control, Beijing, China). All the other viral strains were stored in the laboratory.

### 2.2 Construction of plasmids

To construct the soluble HA protein (rHA), the hemagglutinin (HA) nucleotide sequence of A/Darwin/6/2021 (H3N2) (GISAID accession number: EPI3009100) was codon-optimized according to the codon bias of insect cells and synthesized into pBacPAK9 using GenScript (Nanjing, China) and a 6x His tag was added to the N-terminus of HA. The ectodomain of HA (H) was generated by deleting the transmembrane region. Based on this, we constructed various gene combinations, including HM and HMP, using overlapping polymerase chain reaction (PCR). The PCR cycling parameters consisted of an initial denaturation of 95°C for 10 min, followed by 30 cycles of denaturation at 95°C for 30 s and annealing at 60°C for 30 s, and a final extension of 72°C for 5 min. Three sequential repeats of the M1 (M) sequences, individually or in tandem with the PADRE (P) sequences, were fused to the C-terminus of the HA ectodomain to generate the HM and HMP fusion genes. All genes used were separately fused to the N-terminus of *Helicobacter pylori* ferritin (residues 5-167, GenBank accession number: NP_223316) via a Gly-Gly-Gly-Gly-Gly-Ser linker and a 6x His tag was added to the C-terminus of ferritin, then cloned into pBacPAK9 baculovirus transfer vectors for HA-NP, HM-NP, and HMP-NP nanoparticle production. All constructs were validated using Sanger sequencing.

### 2.3 Expression and purification of the target protein

The proteins were expressed in a baculovirus-insect cell expression system. Briefly, the plasmids were co-transfected into Sf9 cells with linearized bacmid and transfection reagent (Sunma, China) and cultured at 27°C for 6 days to harvest the recombinant baculovirus virus (rBV). And the rBV was passaged to the 3^rd^ generation, then the rBV was inoculated with 1 multiplicity of infection (MOI) into 2.0×10^6^ cells/mL Hi5 cells to express the recombinant protein. After 48 h of incubation, the nanoparticle proteins were secreted into the cell culture supernatant, and the supernatant was collected by centrifugation at 10,000 rpm for 20 min. The rHA is plasma membrane protein, the cells were obtained via centrifugation at 10,000 rpm for 20 min, and HA was extracted from plasma membrane with non-ionic detergent which containing 25 mM phosphate buffer (PB), 150 mM NaCl, 1% Triton X-100, 2 μg/mL leupeptin, pH 7.4. SDS-PAGE gel electrophoresis and western blot were performed to determine the target proteins expression yield. Then the supernatants were replaced with phosphate buffer (25 mM PB, 150 mM NaCl, pH 7.4) via cross-flow ultrafiltration, loaded onto a NTA column (Bestchrom, China), and eluted with washing buffer (25 mM PB, 150 mM NaCl, 500 mM imidazole, pH 7.4). After overnight dialysis, the purified proteins were buffer exchanged with preservation buffer (25 mM PB, 150 mM NaCl, pH 7.4) and filtered through a 0.22-μm membrane, and the protein concentration was determined using a BCA protein assay kit (Thermo, USA) as directed by the manufacturer.

### 2.4 Characterization of the nanoparticles

The purified proteins were analyzed using 10% reducing sodium dodecyl sulfate–polyacrylamide gel electrophoresis (SDS–PAGE), followed by western blotting with an anti-His tag monoclonal antibody (Biodragon, China). Western blotting was performed using enhanced chemiluminescence (ECL) chromogenic solution (Dining, China).

The size and shape of the purified nanoparticles were determined using dynamic light scattering (DLS) and transmission electron microscopy (TEM). For the DLS analysis, the purified nanoparticles were detected using a Zetasizer Nano ZS ZEN3600 (Malvern Instruments Ltd., UK). For TEM analysis, the purified nanoparticle (10 μL) was added dropwise to carbon-coated copper grids for 2 min, followed by staining with 2% phosphotungstic acid for another 2 min. Upon drying, images were recorded using an HT7800 microscope (Hitachi, Japan) at 80 kV.

### 2.5 Mouse immunization and viral challenge

Female BALB/c mice aged 6–8 weeks were randomly divided into 10 groups (n = 15) and immunized intramuscularly (i.m.) (200 μL) or intranasally (i.n.) (50 μL) at a dose of 15 μg of rHA, HA-NP, HM-NP, or HMP-NP protein; and booster immunization was performed on day 28 of vaccination. For the positive control (PC) mice, the same immunization procedures were used, and the mice were i.m. immunized with a quadrivalent inactivated influenza vaccine containing 15 μg HA of each type of influenza strains recommended by WHO in the 2022-2023 north hemisphere influenza season. Negative control (NC) mice were i.m. immunized with 200 μL of PBS.

Primary and booster serum samples were collected 21 and 49 days after immunization, respectively (n = 5). Nasal or lung lavage fluid (n = 3–4) were collected by washing the nostrils and lungs with 1 mL of pre-cooled sterile PBS three weeks after boosting the immunization, and were centrifuged at 12,000 rpm for 3 min. The spleens of immunized mice (n = 4) were isolated after 3 weeks of booster immunization. Lymphocytes were isolated from spleens using mouse 1× Lymphocyte Isolation Medium (7211011, Dakovi Biotech, China). After 4 weeks of booster immunization, mice were challenged intranasally with 15×LD50 of H3N2, 10×LD50 of H1N1, or 10×LD50 of H9N2 influenza strains in 30 μL of PBS. Five days after infection, the mice (n = 4) were sacrificed and lung tissues were isolated for the determination of lung viral titers. Weight loss and survival rates were recorded daily for 2 weeks after infection. Weight loss exceeding 25% was considered a humane endpoint.

### 2.6 Enzyme-linked immunosorbent assay

Antigen-specific IgG, IgA, and IgG subclass antibody titers in serum, nasal washes, and lung wash samples were determined using indirect ELISA. Briefly, ELISA 96-well plates were coated with HA or M1 peptide (1 μg/mL) and blocked with 5% nonfat milk. Serially twofold diluted serum or undiluted nasal and lung wash samples were added as primary antibodies, followed by HRP-conjugated goat anti-mouse IgG, IgA, IgG1, and IgG2a antibodies as secondary antibodies. After washing 3–5 times with PBST, soluble 3,3′,5,5′-tetramethylbenzidine solution (TMB, Beyotime, China) was added and incubated at 37°C for 15 min and the reaction was terminated with 2 M H_2_SO_4_. The optical density was measured at 450 nm using a Prolong DNM-9602 Microplate Reader (Pro-long New Technology Co., Ltd., China), and the highest dilution with an OD_450_ value of more than twice that of the PBS group was regarded as the endpoint of the antibody titer.

### 2.7 HAI and viral neutralization assays

Hemagglutination inhibition (HAI) titers of immunized mouse serum samples were determined as follows: Briefly, a 1% guinea pig erythrocyte suspension and 4 HA_50_ (the highest dilution that causes 50% erythrocytes to agglutinate) units of the A/Darwin/9/2021 (IVR-228) antigen were prepared as hemagglutination working solutions, followed by 4 HA_50_ hemagglutinin calibrations. Before performing the HAI assay, receptor destroying enzyme (RDE; Denka Seiken Co., Ltd., Japan) and sera were mixed in a 3:1 ratio, incubated overnight at 37°C to remove nonspecific inhibitors, and heated at 56°C for 30 min to inactivate the remaining RDE. Finally, concentrated guinea pig erythrocytes were added to the RDE-treated sera at a 1:20 volume ratio to remove non-specific agglutinins. The sera to be examined were diluted to different multiplicities with saline and 4 HA_50_ hemagglutination working solutions were added. After thorough mixing, the solutions were incubated at 37°C for 15 min, and 1% guinea pig erythrocyte suspension was added. The solutions were placed at 20–30°C and measured after 20–40 min. The HAI endpoint titer was expressed as a reciprocal of the highest serum dilution that inhibited virus hemagglutination of 50% erythrocytes.

The median TCID_50_ values of the H1N1, H3N2, and H9N2 viruses were determined using the Reed and Muench method. Heat-inactivated (56°C for 30 min) mouse sera were serially diluted by two-fold in 50 μL DMEM and mixed with 100-fold TCID_50_ of virus in DMEM (50 μL) at 37°C for 2 h. After incubation, the mixture was added to MDCK cells (1.5×10^5^/mL, 100 μL/well, in 2 μg/mL TPCK-trypsin) in a 96-well plate and incubated at 37°C for 3 d. Cytopathic effects were recorded every day, and cell supernatants were collected for hemagglutination assays after 3 days of incubation. Neutralizing antibody titers were finally calculated using the Reed-Muench method.

### 2.8 Cytokine detection assay

Intracellular cytokine levels in the spleen were detected by flow cytometry. Briefly, the spleens of mice were collected 21 days after booster immunization, and lymphocytes were obtained using a mouse 1× lymphocyte separation medium. Lymphocyte suspensions (2×10^6^ cells/mL, 1 mL/well) were inoculated into 24-well plates and stimulated with H3 peptide pools, which along with 1 μL protein transport inhibitor (BFA, BD, USA), for 5-6 h at 37°C. Stimulated lymphocytes were harvested and stained with the live/dead stain (fixable viability stain 620, BD, USA) for 15 min at room temperature, followed by centrifugation at 600 × g for 5 min and discarding the supernatant. Subsequently, 50 μL of Fc blocker (BD, USA) was added to each sample and incubated at 4°C for 10 min. After incubation, cells were collected and stained with APC-Cy7-antimouse-CD3 (BD, USA), PerCP-Cy5.5-antimouse-CD4 (BD, USA) and AmCyan-anti-CD8α (BD, USA) antibodies for 30 min at 4°C. After cell surface staining, the supernatant was discarded after centrifugation, the pellet was washed several times, and cell fixation and membrane breaking were performed by adding a fixation/permeabilization solution (BD, USA). Then, the cells were stained for intracellular cytokines with FITC-conjugated rat anti-mouse IFN-γ (BD, USA) and PE-Cy™7-conjugated rat anti-mouse IL-4 (BD, USA) monoclonal antibodies and incubated at room temperature for 40 min. After washing and resuspending in flow cytometry staining buffer, the stained cells were detected using a BD FACSAria™III flow cytometer and analyzed by FlowJo software.

### 2.9 Determination of lung virus titers

To determine H1N1, H3N2, and H9N2 influenza viral loads in lung tissues, lung tissue samples were weighed and homogenized in 1 mL of pre-cooled PBS, and the supernatants were cleared via centrifugation at 12,000 rpm for 5 min at 4°C. Total viral RNA was extracted from the supernatants using a viral genome DNA/RNA extraction kit (TIANGEN, China), reverse transcribed to cDNA using a reverse transcription kit (TransGen, China), and the copy number of the viral genome was detected using real-time fluorescence quantitative PCR (qPCR).

### 2.10 Statistical analysis

All data were analyzed using GraphPad Prism 8.0 and represented as means ± SEM. The unpaired t-test was used to estimate the statistical significance of differences between two groups. One-way ANOVA with Tukey’s multiple comparison test was used to compare the statistical significance between multiple groups. P < 0.05 was considered to be statistically significant.

## 3 Results

### 3.1 Generation and characterization of HMP-NP nanovaccines

**Figure 1:**
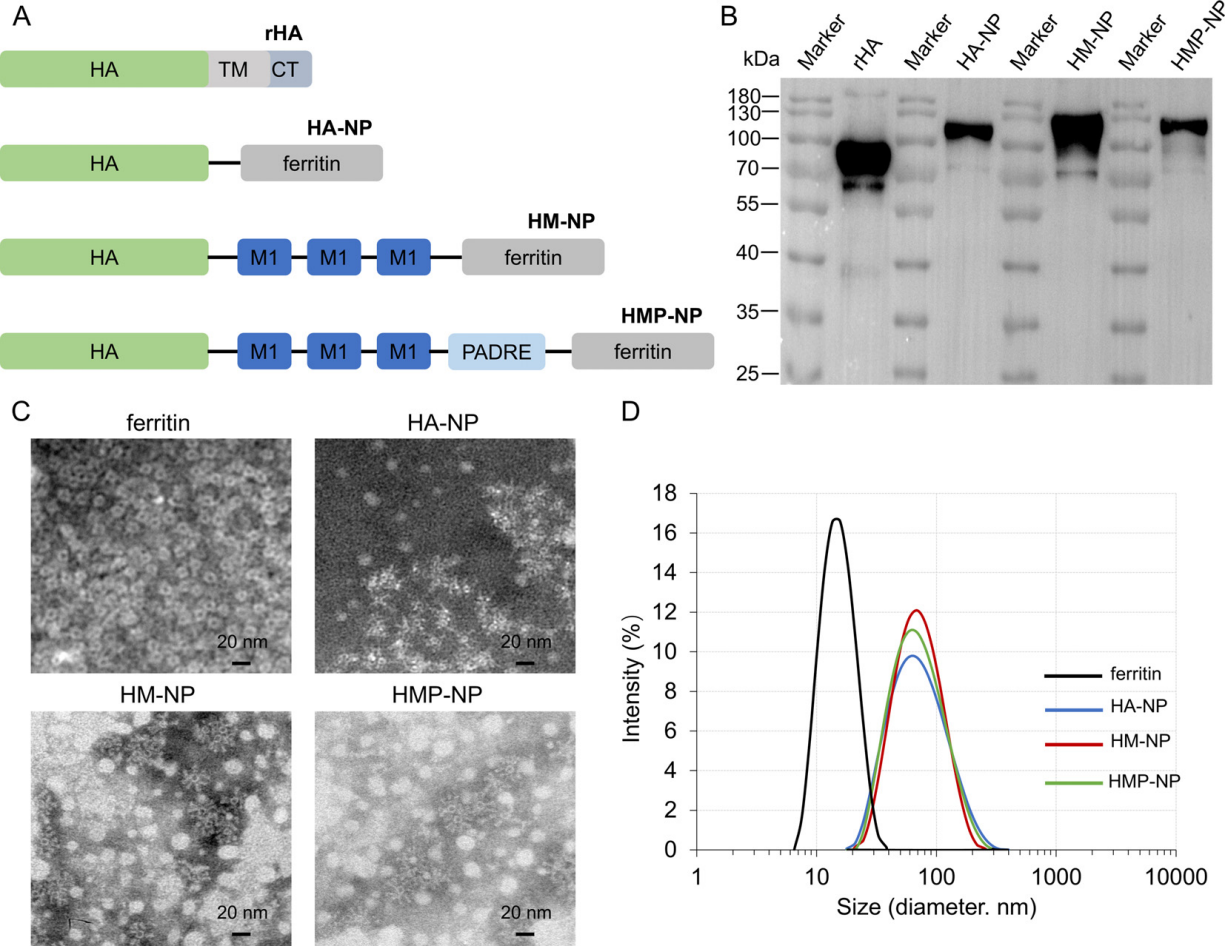
Immunogen design and characterization of nanoparticles. **(A)** Cartoon models of the immunogens construction: HA (H), M1 (M), and PADRE (P) were fused individually or in tandem to the N-terminus of ferritin to generate HA-NP, HM-NP and HMP-NP. **(B)** Western blot analysis of the purified proteins. **(C)** Negative-stain transmission electron microscopy images of HA-NP, HM-NP, and HMP-NP. Scale bar = 20 nm. **(D)** Dynamic light scattering analysis of the ferritin nanoparticles HA-NP, HM-NP and HMP-NP.

To promote the immunogenicity of influenza vaccines, the ectodomain of the H3N2 (A/Darwin/6/2021) virus HA protein, three sequential repeats of M1 epitopes, and the universal T-cell epitope PADRE were fused to the N-terminal of ferritin in tandem to generate HMP-NP (**Figure 1A**). The fusion protein was 6×His-tagged at the C-terminus, expressed in a baculovirus-insect cell expression system, and purified using Ni-affinity chromatography. The molecular weight of the recombinant HMP-NP was determined using western blot, which revealed a major band with a molecular mass of 100-130 Kd (**Figure 1B**). TEM revealed that ferritin alone produced smooth spherical particles, whereas HMP-NPs displayed distinct spikes extending from the spherical core (**Figure 1C**). DLS results showed that the particle size of ferritin alone was approximately 15 nm, and the diameter of the HMP-NPs was approximately 55 nm (**Figure 1D**). In addition, to test whether the HMP-NPs were optimally designed, different nanoparticles were designed to serve as a control group for subsequent immunization efficacy experiments, including HA-NP, HM-NP, and soluble HA protein rHA.

### 3.2 Evaluation of anti-influenza humoral immune responses

**Figure 2:**
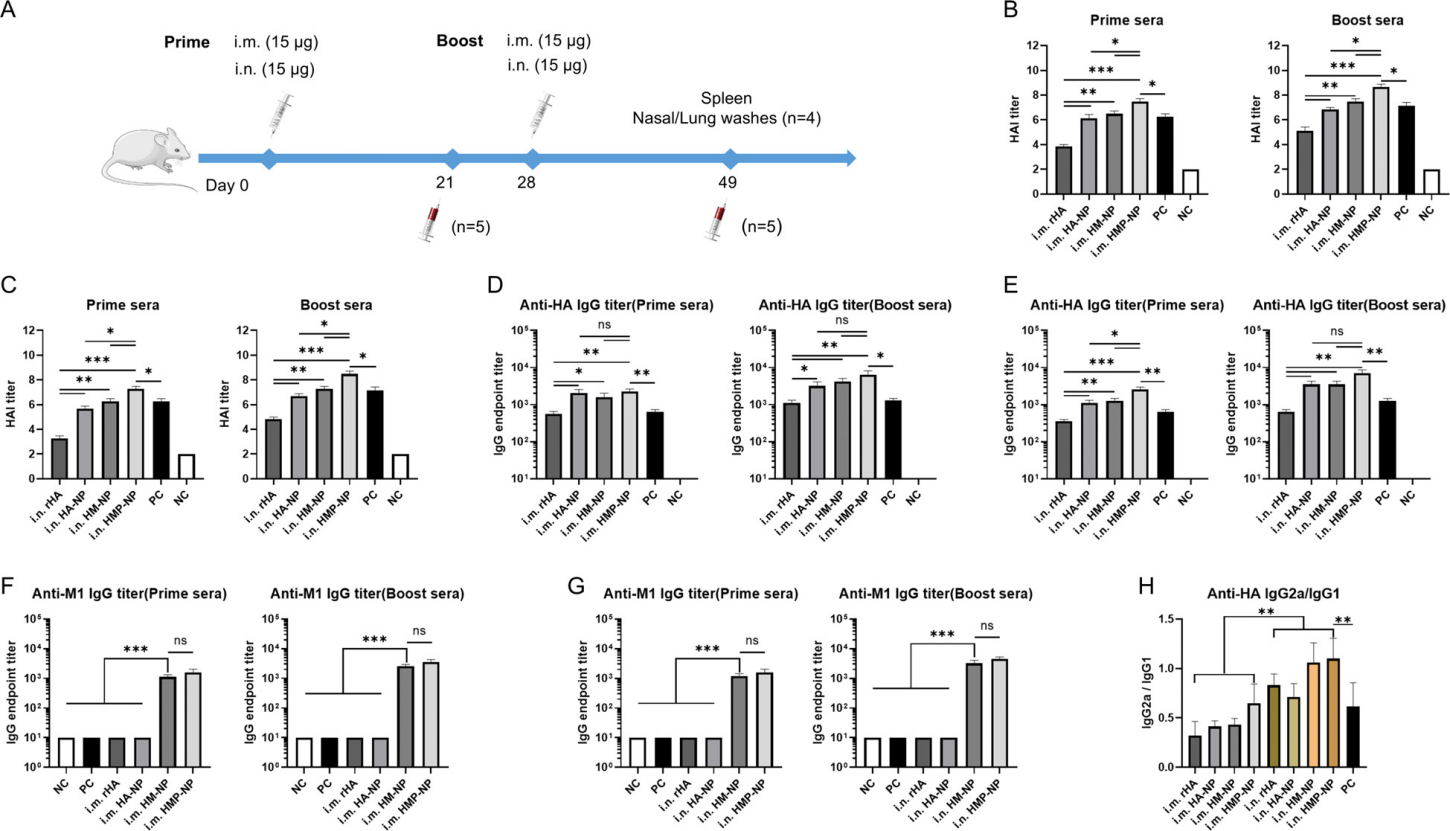
HMP-NP nanoparticles induced robust humoral immune responses in mice. **(A)** Schematic of immunization and sample collection. BALB/c mice were immunized with rHA, HA-NP, HM-NP or HMP-NP via intramuscular (i.m.) or intranasal (i.n.) routes, as indicated. The mice sera were collected on days 21 and 49 and were measured for HAI titers, anti-HA and -M1 IgG antibody levels **(B)** HAI titers in prime and boost sera of i.m.-immunized mice. **(C)** HAI titers in prime and boost sera of i.n.-immunized mice. **(D, E)** HA-specific IgG endpoint titers in prime and boost sera. **(F, G)** M1-specific IgG endpoint titers in prime and boost sera. **(H)** The ratio of HA-specific IgG2a/IgG1 in sera. The data are expressed as the means ± SEM. (n=5; ns, not significant, *p < 0.05, **p < 0.01, ***p < 0.001).

To test whether the above ferritin-based nanoparticle vaccine candidates could induce specific immune responses against influenza HA and M1, 15 μg of the vaccines was i.m. or i.n. administered to BALB/c mice on day 0 and day 28, respectively (**Figure 2A**). The mice were monitored daily for potential adverse effects and body weight was measured for one week after vaccination, and no significant changes were detected. One week after immunization, the lungs of mice were collected for pathological analysis, which showed no significant pathological changes in the lungs (**Supplementary Figure S1**). Sera from immunized mice were collected on days 21 and 49, and HAI titers were determined using a hemagglutination inhibition assay and anti-HA or -M1 antibody levels were measured using antigen-coated ELISA. The results showed that: (i) one dose of nanoparticle vaccines induced significant high HAI titers, anti-HA and -M1 specific antibody titers in the prime sera. Interestingly, three weeks following boosting immunization, the mice immunized with nanoparticle vaccines had small increase in HAI titers (**Figures 2B–G**), indicating that the levels of antigen-specific antibodies in mice have reached the peak after a single dose of nanoparticle vaccine; (ii) i.m. administration induced higher HAI titers and anti-HA specific antibody titers than i.n. administration in both the prime sera and the boost sera, but this increase was not statistically significant (**Figures 2B–E**); and (iii) regardless of the administration route, the HMP-NP nanoparticle vaccine induced much higher HAI titers compared to those of the other vaccines (**Figures 2B, C**), whereas the levels of anti-HA specific antibodies were comparable in the HA-NP, HM-NP and HMP-NP nanoparticle vaccine groups but were higher than those in the rHA and PC groups (**Figures 2D, E**); (iv) HM-NP and HMP-NP induced higher anti-M1 IgG titers than the other vaccines, while the difference was not significant between the HM-NP and HMP-NP groups (**Figures 2F, G**); (v) the results of the detection of the antibody IgG subtypes indicated that i.m. administration of all vaccine candidates induced lower IgG2a antibody levels than IgG1, indicating Th2-favored responses, whereas i.n. administration of the HM-NP and HMP-NP nanoparticle vaccines showed a balanced immune response with IgG1 antibody levels comparable to that of IgG 2a (**Figure 2H**).

**Figure 3:**
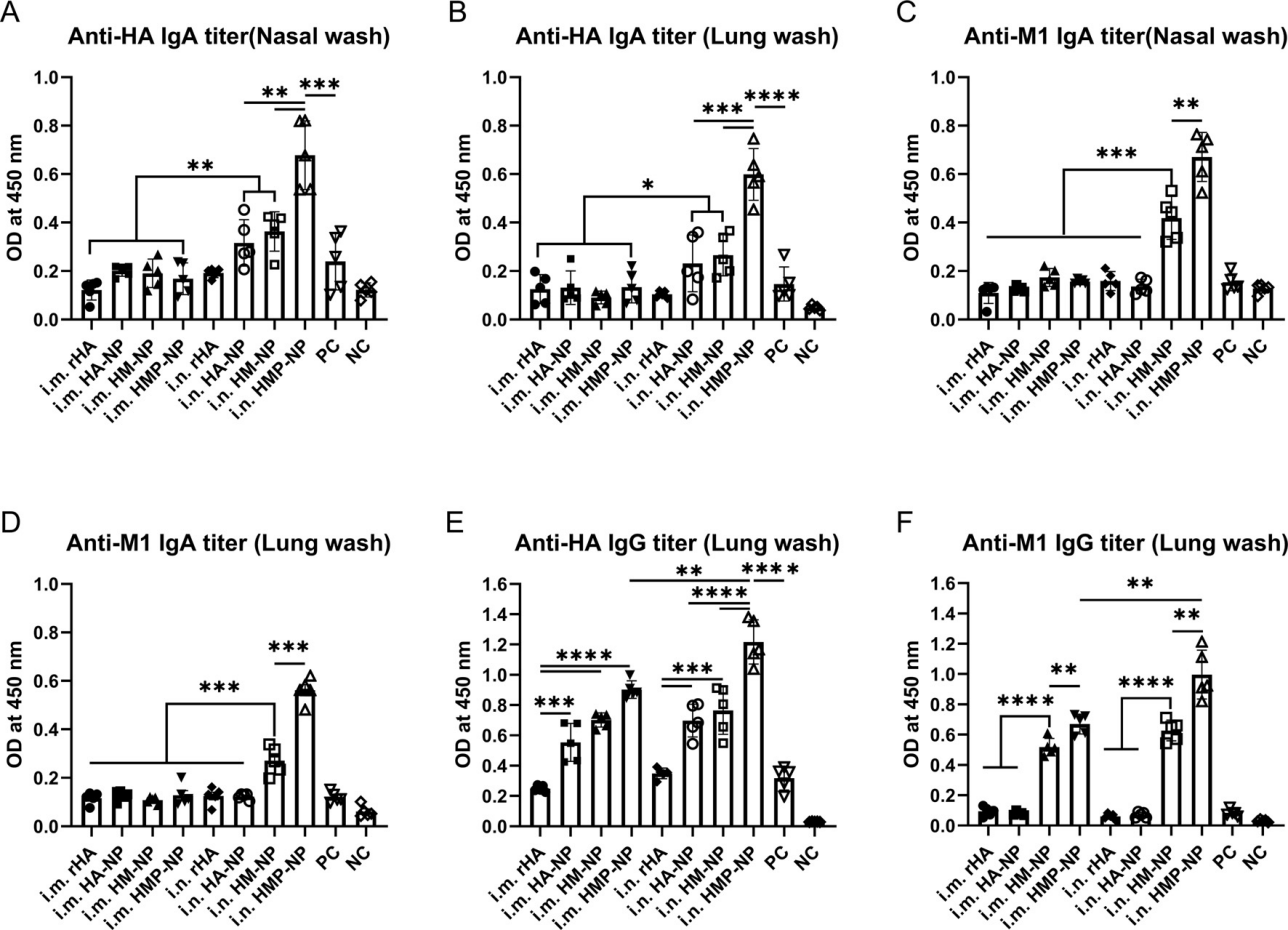
Induction of local mucosal immune responses following immunization with HMP-NP nanoparticles in mice. **(A)** Anti-HA IgA levels in nasal washes. **(B)** Anti-HA IgA levels in lung washes. **(C)** Anti-M1 IgA levels in nasal washes. **(D)** Anti-M1 IgA levels in lung washes. **(E)** Anti-HA IgG levels in lung washes. **(F)** Anti-M1 IgG levels in lung washes. The data are expressed as the means ± SEM. (n=4; *p < 0.05, **p < 0.01, ***p < 0.001, ****p < 0.0001).

Cross-reactive sIgAs provide broad protection against heterologous and heterosubtypic influenza viruses (40). To evaluate the levels of mucosal IgA and IgG antibodies on the respiratory tract surfaces after vaccination, we collected nasal and lung washes from mice 21 days after booster immunization. The assay results showed that (i) i.n. administration of all vaccine candidates induced higher levels of anti-HA and -M1 sIgA antibodies in the nasal and lung wash samples compared to those induced by i.m. administration (**Figures 3A–D**); (ii) HA-NP, HM-NP, and HMP-NP nanoparticle vaccines induced higher anti-HA IgG titers in the lung washes than rHA and PC groups regardless of the administration route (**Figure 3E**); and (iii) HMP-NP induced the highest levels of sIgA and IgG antibodies compared to those of other vaccines; (iv) HM-NP and HMP-NP elicited higher anti-M1 IgG titers in lung washes compared to the other vaccines (**Figure 3F**). These observations indicate that immunization with the HMP-NP vaccine induced efficient anti-HA immune responses and the i.n. administration route induced potent mucosal immune responses.

### 3.3 Vaccination with HMP-NP nanovaccine-induced potent cross-neutralizing antibodies

**Figure 4:**
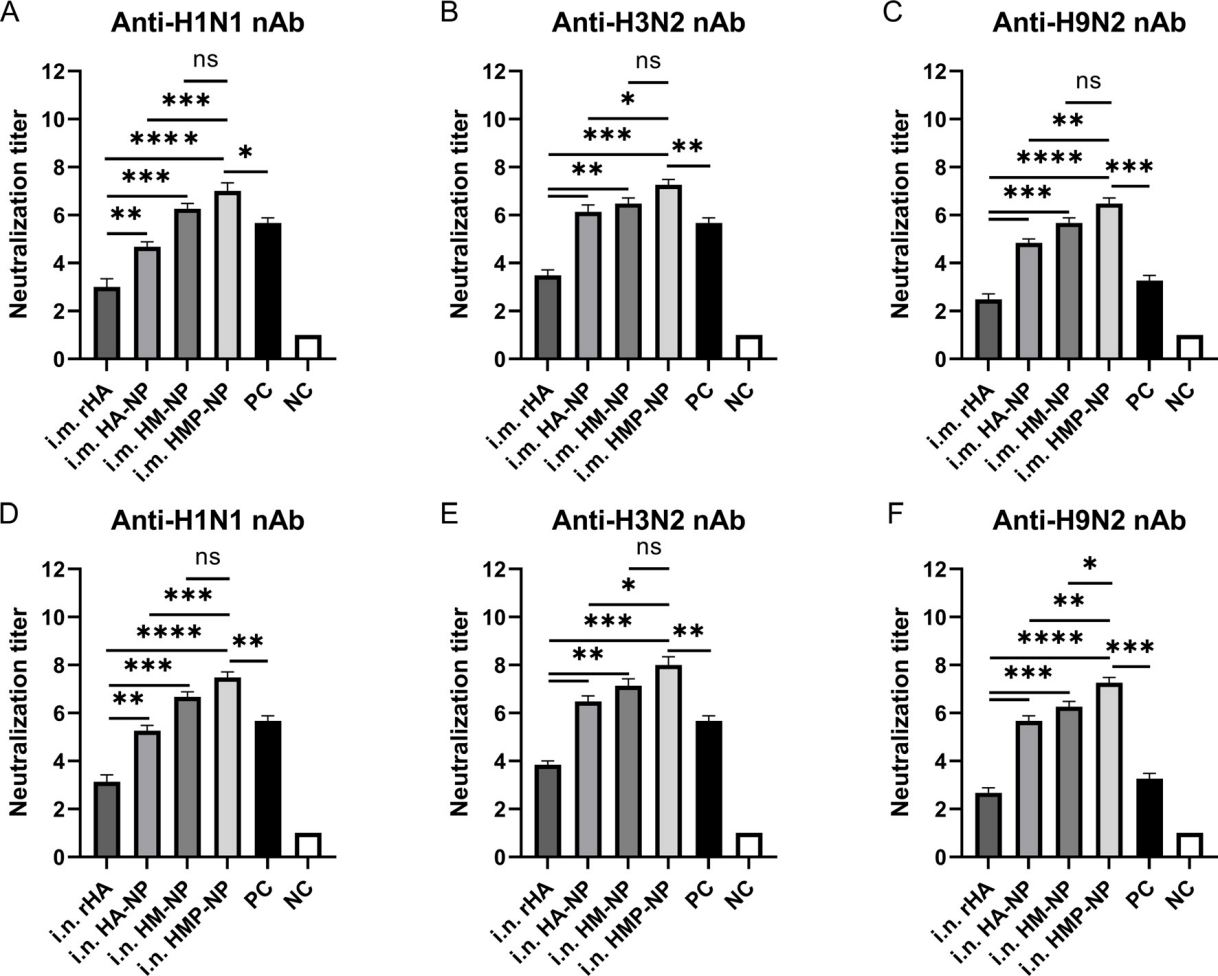
Neutralizing antibody (nAb) titers in mice immunized with HMP-NP nanoparticles. **(A–C)** nAb titers in intramuscular-immunized mice sera. **(A)** Anti-H1N1 nAb titers. **(B)** Anti-H3N2 nAb titers. **(C)** Anti-H9N2 nAb titers. **(D–F)** nAb titers in intranasal-immunized mice sera. **(D)** Anti-H1N1 nAb titers. **(E)** Anti-H3N2 nAb titers. **(F)** Anti-H9N2 nAb titers. The data are expressed as the means ± SEM. (n=5; ns, not significant, *p < 0.05, **p < 0.01, ***p < 0.001, ****p < 0.0001).

We assessed whether the antibodies produced by the ferritin-based nanoparticle vaccines possessed neutralizing activity by performing neutralization assays using different influenza strains, including A/Puerto Rico/8/34 (H1N1), CVCC AV1520 (H3N2), and A/Chicken/Jiangsu/7/2002 (H9N2). As shown in **Figure 4**, the results showed that vaccination with the HMP-NP nanoparticle vaccine elicited the highest levels of neutralizing antibodies (nAbs) against homologous strains of the H3N2 virus or heterologous strains of the H1N1 and H9N2 viruses. In contrast, rHA showed low neutralizing activity (**Figures 4A–F**), which was consistent with the low HAI titers in the immunized sera (**Figures 2B, C**). Vaccination with HA-NP and HM-NP induced higher levels of cross-nAbs than rHA-soluble HA protein, and the difference between the levels of nAb induced by HM-NP and HMP-NP was not significant. These results suggested that nanoparticle vaccines are the most efficient at producing nAbs. Notably, nAb titers were higher in the i.n. group than those in the i.m. group, although this increase was not statistically significant. The sera of vaccine-immunized mice in the PC group contained higher levels of nAbs against homologous strains of the H1N1 and H3N2 viruses (**Figures 4A, B**), whereas those against the heterologous strain of the H9N2 virus were lower (**Figure 4C**). In summary, among the four vaccine candidates, the HMP-NP vaccine elicited the highest titer of nAbs against multiple influenza virus infections.

### 3.4 HMP-NP nanoparticles induced strong cellular immune responses

**Figure 5:**
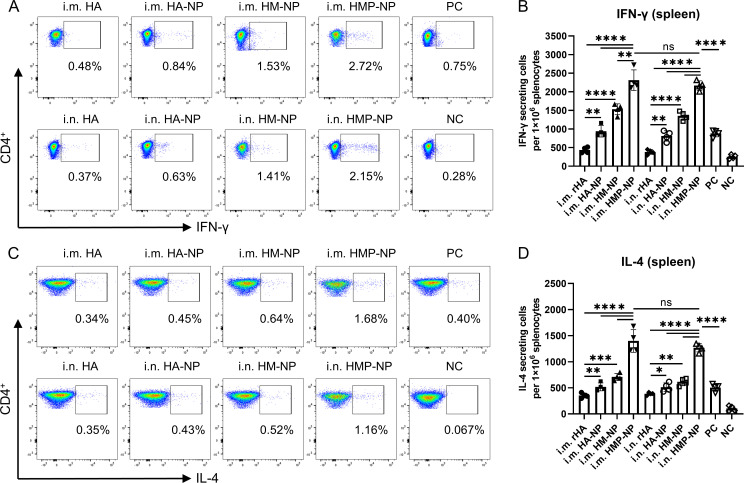
Cellular immune responses. **(A)** Representative flow cytometry analysis results of the percentages of IFN-γ. **(B)** Number of IFN-γ-secreting CD4^+^ T cells. **(C)** Representative flow cytometry analysis results of the IL-4 levels. **(D)** Number of IL-4-secreting CD4^+^ T cells. The data are expressed as the means ± SEM. (n=4; ns, not significant, *p < 0.05, **p < 0.01, ***p < 0.001, ****p < 0.0001).

Cellular immunity plays an important role in fighting influenza virus infections (41). To evaluate whether HMP-NPs induced HA-specific cellular immune responses, the spleens of mice were collected to isolate splenic lymphocyte suspensions 21 days after booster immunization. Splenic lymphocytes from control and immunized mice were stimulated with 10 μg/ml HA peptide, and the number of IFN-γ and IL-4 secreting cells were analyzed using intracellular cytokine staining combined with flow cytometry. Gating strategies were used for effective flow cytometry data analysis of T lymphocytes (**Supplementary Figure S2**). The results showed that mice immunized with nanoparticles generated more IFN-γ and IL-4-secreting cells than mice in the rHA soluble HA protein or PC groups, regardless of the administration route. Importantly, mice immunized with HMP-NP nanoparticles generated significantly more IFN-γ (**Figures 5A, B**) and IL-4-secreting cells (**Figures 5C, D**). These findings demonstrate that the ferritin-based HMP-NP vaccine possesses good immunogenicity to elicit potent cellular immune responses.

### 3.5 HMP-NP nanoparticles provided cross-protection against influenza virus challenge

**Figure 6:**
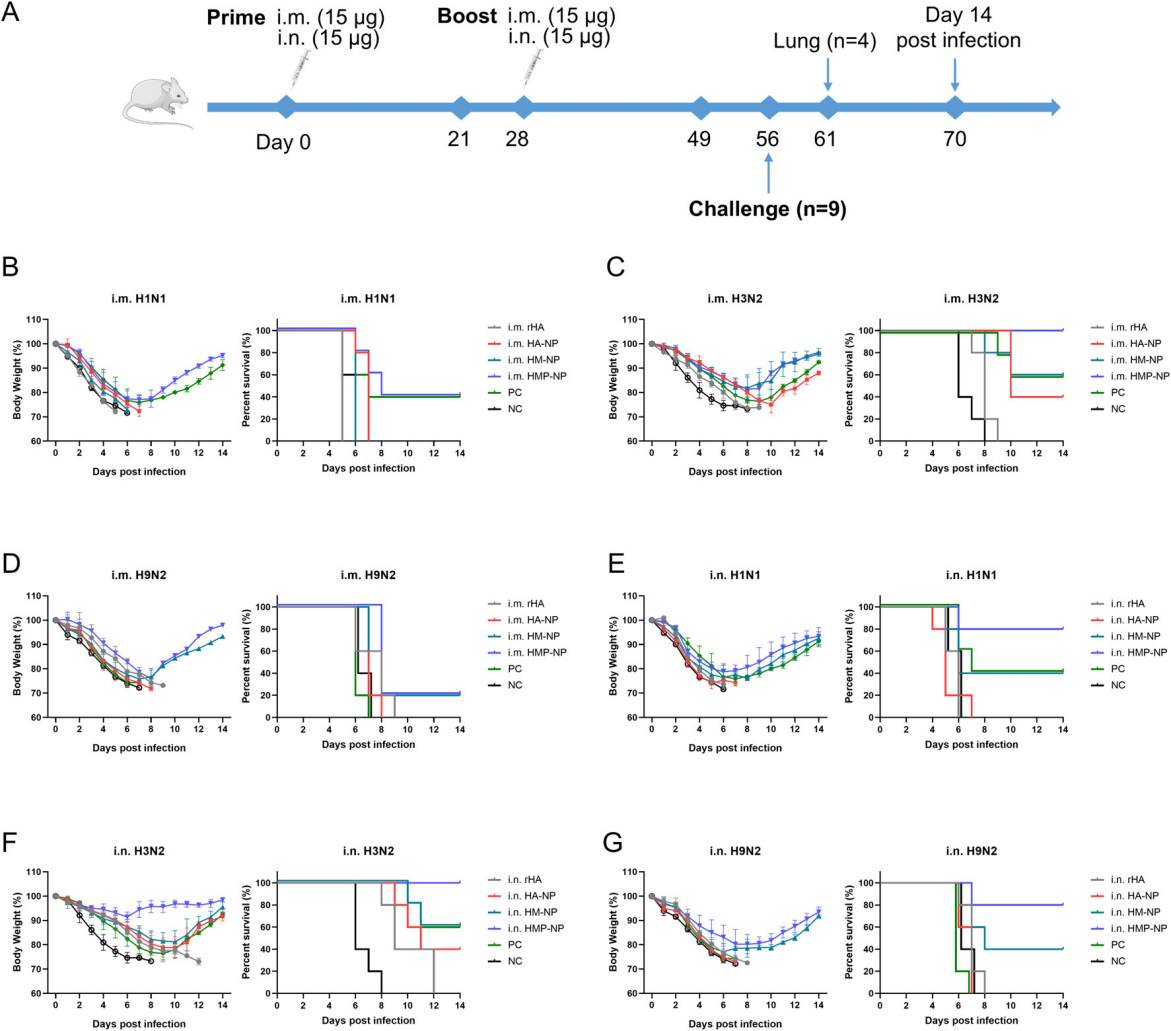
Protective efficacy of HMP-NP nanoparticles against IAV challenge in mice. **(A)** Experimental schema. **(B-D)** Body weight changes and survival rates of intramuscular-immunized mice were intranasally challenged with **(B)** A/PR/8 (H1N1), **(C)** CVCC AV1520 (H3N2), or **(D)** A/Chicken/Jiangsu/7/2002 (H9N2) 4 weeks after booster immunization (n=5). **(E-G)** Body weight changes and survival rates of intranasal-immunized mice were challenged intranasally with **(E)** A/PR/8 (H1N1), **(F)** CVCC AV1520 (H3N2) or **(G)** A/Chicken/Jiangsu/7/2002 (H9N2) for 4 weeks after booster immunization (n=5).

To assess the protective efficacy of each vaccine candidate, all mice were immunized twice at 4-week intervals via i.m. or i.n. administration. Twenty-eight days after booster immunization, all mice were challenged with different influenza strains, including a 15× median lethal dose (LD50) of A/PR/8 (H1N1), 10× LD50 of H3N2 (CVCC AV1520), or 10× LD50 of A/Chicken/Jiangsu/7/2002 (H9N2). The body weight and survival rates of mice were monitored daily for 14 days. As shown in **Figures 6B–G**, infection with the influenza viruses resulted in a large loss of body weight (over 25%) in the PBS and rHA control mice, and they died within 8-12 days. In contrast, regardless of the administration route, all mice immunized with HMP-NPs survived lethal challenges with the homologous strain H3N2 virus infections and showed slight weight loss (**Figures 6C, F**). In the lethal challenges of infection with heterologous strains of H1N1 and H9N2 viruses, i.m. administration of HMP-NP provided only 40% and 20% partial protection (**Figures 6B, D**), whereas i.n. administration provided 80% protection (**Figures 6E, G**). HA-NPs immunization provided only 40% partial protection against H3N2 virus (**Figures 6C, F**), and no protection against heterologous strains of H1N1 and H9N2 viruses (**Figures 6B, D**). Mice immunized with HM-NP exhibited partial protection with variable efficacy (20%-60% mouse survival) and suffered severe weight loss when challenged with different influenza viruses. In the PC group, 40%-60% of mice survived lethal challenges with the homologous strains of H1N1 or H3N2 viral infections (**Figures 6B, C**), whereas all mice died after lethal challenge with the heterologous strain of H9N2 viral infections (**Figure 6D**).

**Figure 7:**
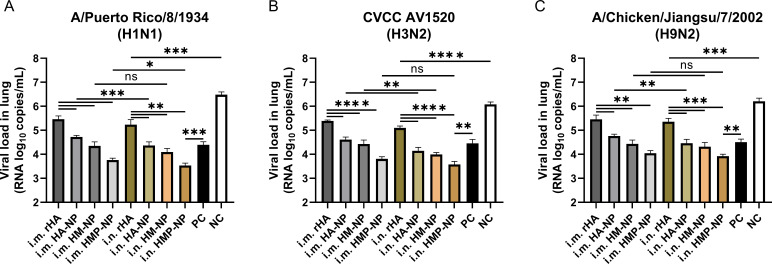
Determination of lung viral titers on day 5 post-infection. **(A)** H1N1 viral titers in the lungs. **(B)** H3N2 viral titers in the lungs. **(C)** H9N2 viral titers in the lungs. The data are expressed as the means ± SEM. (n = 4; ns, not significant, *p < 0.05, **p < 0.01, ***p < 0.001, ****p < 0.0001).

Furthermore, we monitored the viral loads in the lungs of mice 5 days post-challenge. As shown in **Figures 7A–C**, the results showed that the viral loads of H1N1, H3N2, and H9N2 in the PBS control mice reached approximately 3.0×10^6^, 1.2×10^6^ or 1.6×10^6^ copies/mL, respectively, while vaccinated mice in all groups showed lower viral loads. Importantly, the viral loads in the HMP-NP-immunized mice were only approximately 10^3^ copies/mL, indicating that viral replication in the lungs of HMP-NP-immunized mice was greatly suppressed. Mice immunized via the i.n. route showed lower lung viral loads than mice in the i.m. route group, which is consistent with the protective efficiency results, although some data were not statistically significant. In summary, these results provide strong evidence that vaccination with HMP-NPs effectively protected mice from lethal influenza virus challenges, and that intranasal immunization with HMP-NPs could induce potent protection against heterologous influenza viruses.

## 4 Discussion

Influenza is a highly contagious respiratory disease that poses an enormous public health burden worldwide (1, 2). Vaccination remains the most effective and economical strategy for preventing influenza virus infections. However, current seasonal influenza vaccines induce strain-specific immune responses with protective efficacies ranging from 10% to 60%, and are less effective against mismatched pandemic strains (42–45). In this study, we developed a universal vaccine candidate, HMP-NP, by utilizing the ferritin nanoparticle platform, in which the ectodomain of HA, broad-spectrum T-cell epitope M1, and universal T-cell epitope PADRE were fused in tandem with the N-terminus of ferritin, and the fusion proteins self-assembled into nanoparticles. Animal immunization studies indicated that the HMP-NP vaccine can induce potent humoral and cellular immune responses, including the production of nAbs, against different influenza A virus infections and effectively protect mice against lethal H1N1, H3N2, and H9N2 infections.

The current focus of universal vaccine development is to target the relatively conserved regions of the influenza virus and utilize various means of antigen delivery to generate broad-spectrum immune responses (46). External protein-targeting vaccines can provide complete protection against homologous viruses but lack broad-spectrum protection; for example, vaccines based on HA and NA are usually only effective against the same virus subtype (47, 48). Internal protein-targeting vaccines can promote viral clearance by inducing T-cell immune responses, resulting in a broader spectrum of protection (15, 16, 49). Owing to the poor immunogenicity of protein-based vaccines, optimizing antigen delivery conditions or multitarget co-actions is often necessary to improve vaccine immunity.

The selection of suitable vaccine targets, such as the HA ectodomain and the highly conserved M1 epitope, is critical for the development of a universal influenza vaccine. The HA protein is the most important surface glycoprotein of the influenza virus, mediating viral invasion into host cells, and the main target of anti-influenza virus nAbs (8). The M1 protein is the most abundant and conserved protein in viral particles; it induces T-cell immune responses and provides cross-immunoprotection (49). To further increase the immunization effect of the vaccine, we fused the universal T cell epitope PADRE to the antigen as an immunostimulant. Three peptides were fused in tandem to the N-terminus of ferritin and self-assembled into nanoparticles to simulate natural viruses. Animal immunization studies demonstrated that the tri-epitope combination nanoparticles, HMP-NP, were superior to bivalent (HM-NP) and monovalent (HA-NP) nanoparticles in inducing immune responses and cross-reactive protection. Therefore, optimization of antigen delivery conditions and selection of multitarget co-actions are good strategies for the development of a universal influenza vaccine.

The utilization of nanoplatforms to deliver antigens is a promising approach for the development of novel influenza vaccines. In this study, we developed a nanoparticle vaccine, HMP-NPs, without additional adjuvants that induced robust humoral and cellular immune responses and provided protection against homologous and heterologous influenza virus challenges. Previous studies have shown that nanoparticle vaccines are capable of effectively accumulating in lymph nodes, thereby improving immune processing (50–52). Additionally, nanoparticle vaccines are readily taken up by dendritic cells (DCs) and macrophages, which then process and present antigens to CD4 T helper cells, followed by the cooperation of T follicular helper (Tfh) cells and B cells to enhance the antibody responses (53, 54). The high density and structural order of antigens on the surface of nanoparticles facilitates antigen recognition by B-cell receptors (BCRs) and triggers effective cellular and humoral immune responses (55, 56). In addition, nanoparticles have been shown to protect antigens from proteolytic degradation, improve antigen delivery, and prolong antigen presentation by antigen-presenting cells (APCs) (57, 58). Ferritin nanoparticles exhibit viral antigens at a high density on their surfaces to efficiently induce humoral and cellular immune responses and have been widely used in the prevention and treatment of a variety of diseases, such as influenza, COVID-19, hepatitis B, and AIDS (34–37).

Enhancing the potency and breadth of antibody responses is essential for the development of universal influenza vaccines. Regardless of the administration route, mice immunized with the nanoparticle vaccines (HA-NP, HM-NP, or HMP-NP) exhibited enhanced systemic humoral immune responses. We observed significantly higher levels of antigen-specific IgG antibodies, HAI titers, and nAb titers in the mouse immune sera of the nanoparticle groups compared with those of the soluble protein rHA group. Interestingly, we found that the second dose of nanoparticle vaccines in mice did not significantly enhance HAI titers and IgG titers, suggesting further research is required to confirm whether a single dose of nanoparticle vaccine is sufficient to protect against influenza virus infection. Moreover, we detected significantly enhanced cross-reactive antibody titers against the homologous H3N2 virus and heterologous H1N1 and H9N2 viruses. Importantly, the HMP-NP vaccine exhibited the best immunization effect among all the vaccine candidates.

The respiratory mucosa is the first line of defense against pathogens, and an effective mucosal immune response provides cross-protective immunity against various influenza viral infections. Intranasal immunization is a safe and effective method for inducing mucosal immunity and resistance to influenza viruses. Our results showed that both i. m. and i.n. immunization with HMP-NPs induced high levels of HA- and M1-specific IgG antibodies in the lungs of the mice. In the nose and lungs, we observed significantly enhanced sIgA antibody titers following i.n. administration. As sIgA is more broadly reactive than IgG (59), sIgA triggered by i.n. immunization with HMP-NPs plays a crucial role in providing cross-protection against various influenza virus infections.

Rapid T-cell responses following vaccination play a critical role in resistance to influenza virus infection. In this study, we observed high levels of secreted cytokines, including IFN-γ and IL-4, in the splenocytes of mice immunized with the HMP-NP nanoparticle vaccine. IL-4 is a Th2-type cytokine that promotes the proliferation and differentiation of B lymphocytes into antibody-secreting plasma cells. These results were consistent with the observed elevation in antigen-specific IgG levels. IFN-γ can inhibit viral replication and modulate cellular immunity during viral infection, and significantly elevated levels of IFN-γ cytokines contributed to broad protective effects against divergent influenza virus infections following HMP-NP nanoparticle vaccination.

In addition, we investigated the cross-protective ability of the HMP-NP nanovaccine against influenza A virus. The results showed that i.m. or i.n. administration of HMP-NP not only provided complete protection against homologous strains of H3N2 virus challenge but also provided cross-protection against heterologous strains of H1N1 and H9N2 virus challenge to a certain extent; the survival rates in the i.m. route group were 40% and 20%, respectively, and that in the i.n. route group reached 80%. Enhanced antibody and T-cell responses elicited by HMP-NPs in the peripheral circulatory system via i.m. and i.n. administration routes, which contributed to cross-protection in HMP-NPs nanoparticle vaccination. Meanwhile, we also observed significantly enhanced sIgA antibody titers in respiratory mucosa following i.n. administration, which could neutralize influenza virus infectivity during transcytosis in the infected epithelial cells. As an outcome, the mucosal immunity triggered by sIgA conferred the increased cross-protection efficacy. The stronger cross-protection against heterologous influenza viruses induced by i.n. immunization compared to that induced by the i.m. route is largely attributed to the synergistic function of T-cells, systemic and mucosal immune responses. Given the limitations of the virus source, this research only verified the protective effect of HMP-NP influenza nanoparticle vaccine on H1N1, H3N2 and H9N2 strains, and whether it can elicit cross-protection against more types of influenza A viruses or influenza B viruses will be further proven.

Long-term immunity or durability of responses are critical factors for evaluating vaccine effectiveness. Previous studies have shown that nanoparticle vaccines could produce long-lasting protective immune responses (60). In our study, we monitored the antibody level only until the 21th day after booster immunization, and further investigation is needed to verify whether the HMP-NP nanoparticle vaccine can also stimulate longer-lasting immune responses and protection.

## 5 Conclusion

In conclusion, we developed a ferritin-based influenza nanoparticle vaccine, named HMP-NP. In the absence of additional adjuvants, ferritin nanoparticles displaying high-density multi-antigen targets induced strong humoral and cellular immune responses in mice while providing cross-protection against lethal influenza virus challenge. Thus, these results provide proof-of-concept for developing potent universal influenza vaccines using a self-assembled ferritin nanoparticle platform that incorporates multiple antigen targets.

## Data Availability

The original contributions presented in the study are included in the article/supplementary material. Further inquiries can be directed to the corresponding author.
