# Supplementary material for: Adjuvant-free, self−assembling ferritin nanoparticle vaccine coupled with influenza virus hemagglutinin protein carrying M1 and PADRE epitopes elicits cross-protective immune responses
Source: Front Immunol. 2025 Jan 31;16:1519866. doi: 10.3389/fimmu.2025.1519866 (PMC11827429; doi:10.3389/fimmu.2025.1519866)
Supplement: Supplementary file 1 [file DataSheet1.docx]

Supplementary Material

# Supplementary Figures


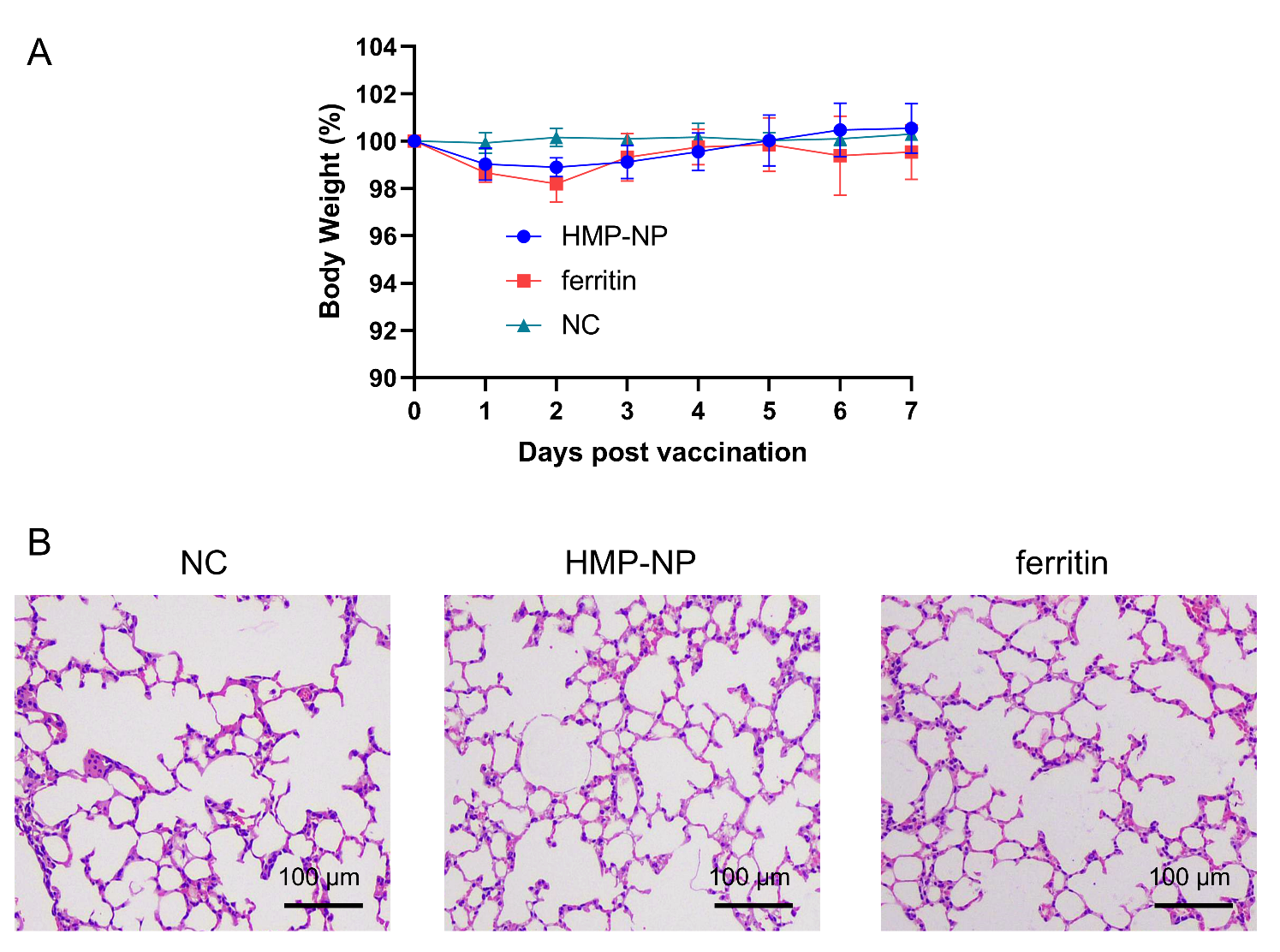


**Supplementary Figure 1.** Safety evaluation post-vaccination with HMP-NP nanoparticles. (A) Body weight changes for 7 days post-vaccination. (B) Lung histological analysis for 7 days post-vaccination.


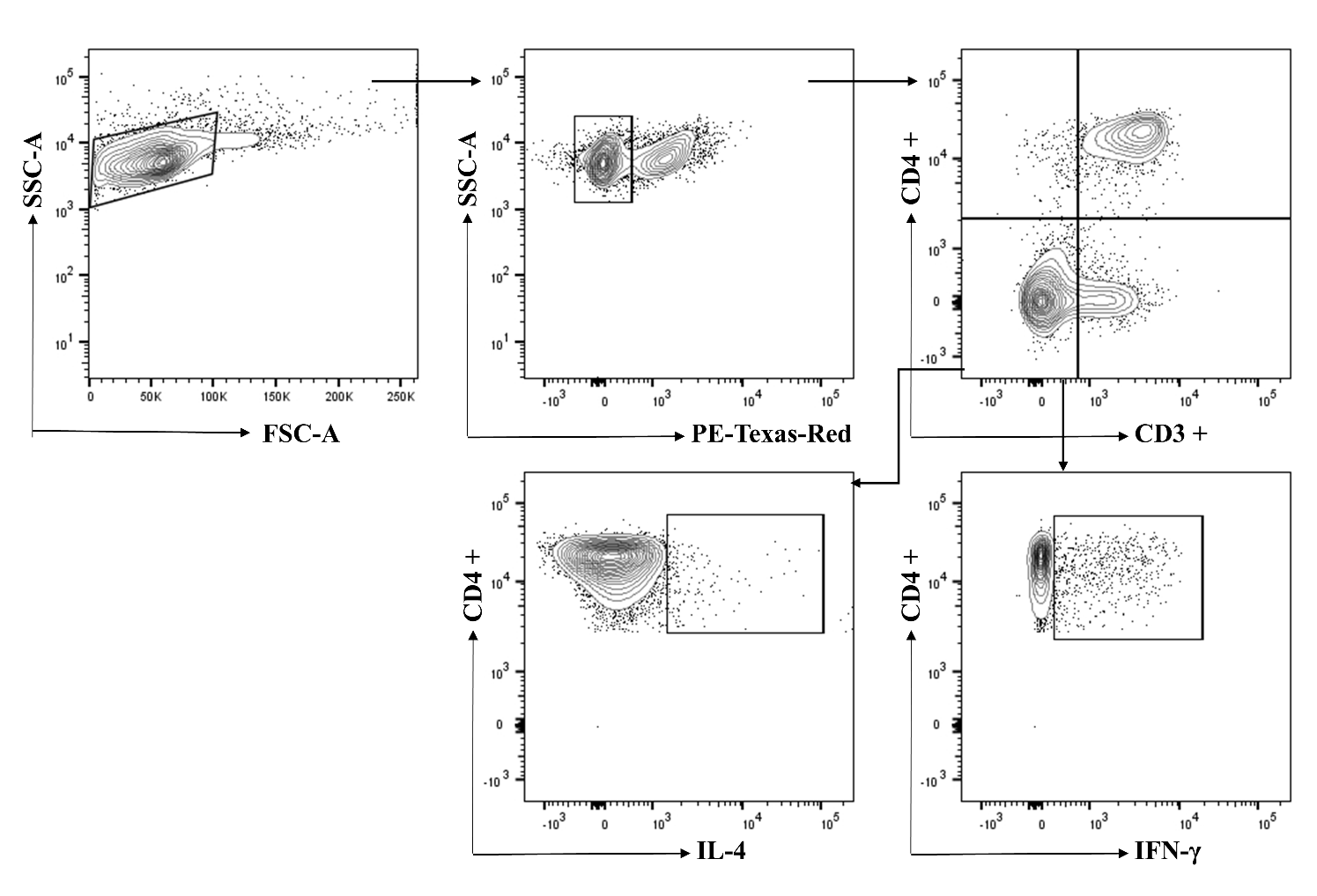


**Supplementary Figure 2.** Gating strategy for flow cytometry analysis of T lymphocytes in the spleen.
